# Effect of *Lactobacillus plantarum* IS-10506 on blood lipopolysaccharide level and immune response in HIV-infected children

**Published:** 2019-04

**Authors:** Alpha Fardah Athiyyah, Herwina Brahmantya, Stephani Dwiastuti, Andy Darma, Dwiyanti Puspitasari, Dominicus Husada, Reza Ranuh, Anang Endaryanto, Ingrid Surono, Subijanto Marto Sudarmo

**Affiliations:** 1 Department of Child Health, Faculty of Medicine, Dr. Soetomo Hospital, Airlangga University, Surabaya, Indonesia; 2 Department of Food Technology, Faculty of Engineering, Bina Nusantara University, Jakarta, Indonesia

**Keywords:** Probiotics, *Lactobacillus plantarum* IS-10506, HIV infected children, Blood lipopolysaccharide, Immune responses

## Abstract

**Background and Objectives:**

HIV enteropathy may cause disruption of the intestinal barrier, leading to a loss of CD4+ T cells, increased intestinal permeability, and microbial translocation. *Lactobacillus plantarum* IS-10506 has the ability to improve gut barrier function. This study investigated the effect of *L. plantarum* IS-10506 on a number of biomarkers of enteropathy-related damage in HIV-infected paediatric patients undergoing antiretroviral therapy (ARV).

**Materials and Methods:**

A randomized, double-blind, placebo-controlled study was conducted on 2–18 year-old children, diagnosed as HIV infected according to the WHO 2007 criteria who had received ARV for ≥ 6 months. Subjects were excluded if ARV therapy was discontinued or the patients took probiotics ≥ 2 weeks prior to the study or during the study period. Subjects were randomized into a probiotic group and placebo group. The probiotic group received *L. plantarum* IS-10506 2.86 × 10^10^ cfu/day for 6 days. Blood lipopolysaccharide (LPS) level, serum CD4+ T cell count, serum CD8+ T cell count, CD4+/CD8+ T cell ratio, and faecal sIgA level were assessed as biomarkers.

**Results:**

Twenty-one subjects completed this study. The blood LPS level decreased significantly in the probiotic group (*p* = 0.001). There was no significant difference in absolute CD4+ T cell count, percent CD4+ cells, absolute CD8+ T cell count, CD4+/CD8+ T cell ratio, or faecal sIgA. No serious adverse events were reported.

**Conclusion:**

The probiotic *L. plantarum* IS-10506 reduced the blood LPS level but showed no effect on the humoral mucosa and systemic immune response in HIV-infected children undergoing ARV therapy.

## INTRODUCTION

Human immunodeficiency virus (HIV) is a virus that infects and kills CD4+ T cells and plays an important role in the immune response to the invasion of pathogenic microorganisms (1). In 2016, there were up to 1.8 million newly infected patients, consisting of 1.7 million adults and 160 thousand children under 15 years of age (2).

Enteropathy commonly occurs following HIV infection (3). Enteropathy can cause microbial translocation from the intestinal lumen to the systemic blood circulation, thus stimulating chronic immune activation (4). The degree of this bacterial translocation can be reflected by the blood lipopolysaccharide (LPS) level, as LPS is the main component of the cell wall of Gram-negative bacteria, the predominant bacteria in the gastrointestinal tract (5, 6). A previous study showed that there is a significant increase in the plasma LPS level in patients with chronic HIV infection, compared with those without HIV (3). Although antiretroviral (ARV) therapy can suppress viral replication in HIV patients, it does not facilitate complete improvement and is unable to repair intestinal barrier injury, which causes microbial translocation and eventually stimulates chronic immune activation (7–9).

Probiotics are microorganisms that, in optimal amounts, can provide health benefits to their host (10) mainly because of their ability to enhance the mucosal immune response, repair intestinal barrier tight junctions, and help reduce the colonization and translocation of pathogenic microbes (11). Prior research has found that supplementation of probiotics in HIV-infected patients treated with ARV improved the health of the digestive system and patients’ long-term prognosis (8).

*Lactobacillus plantarum* IS-10506 is a probiotic strain isolated from Indonesian fermented water buffalo milk known as “dadih “(12). Probiotic supplementation of *L. plantarum* IS-10506 and IS-20506 has been shown to inhibit apoptosis and intestinal brush border epithelial cell damage by inhibiting nuclear factor kappa B (NF-κB) and downregulating TNF-α receptor 1 (TNFR1) (13). The supplementation contributed to the repair of intestinal brush border epithelial damage after *E. coli* LPS exposure (13). We therefore investigated the effect of oral supplementation of *L. plantarum* IS-1050 on the amount of microbial translocation (as shown by the blood LPS level), serum CD4+ T cell counts, CD8+ T cell counts, CD4+/CD8+ T cell ratio (which reflects HIV infection progression), and faecal secretory IgA (a humoral immune parameter) in HIV-infected children receiving ARV therapy.

## MATERIALS AND METHODS

### Study design.

We designed a randomized double-blind placebo-controlled study (clinical trial number TCTR20180525003 from Thai Clinical Trial Registry), which was conducted from December 2012 to March 2013. An ethical clearance certificate was issued by the Dr. Soetomo Hospital Research Ethical Committee, with certificate number 217/Panke KKE/IX/2012. Subjects were recruited from the Intermediate Care of Infectious Disease Unit (ICI-DU) outpatient clinic in Dr. Soetomo General Hospital, Surabaya, Indonesia.

Randomization was done independently by the pharmacist using simple random sampling. Subjects were allocated to two groups: a probiotic group and a placebo group. Those in the probiotic group received 700 mg of microencapsulated probiotic with a dose of 2.86 × 10^10^ cfu/day, given orally once daily for 6 weeks. The placebo group received 700 mg powdered maltodextrin, packaged and flavoured in the same manner as the probiotic, given once a day for 6 weeks. Both groups continued the standard ARV therapy during the study. The blood LPS level, absolute CD4+ T cell count, percent CD4, absolute CD8+ T cell count, CD4+/CD8+ ratio, and faecal sIgA were measured before and after treatment.

### Subjects.

Inclusion criteria were: (1) 2–18 years of age; (2) HIV infected, based on WHO 2007 immunological criteria for diagnosing HIV in children; (a) CD4 count less than 350 per mm3 of blood in children 5 years or older, and/or (b) %CD4 < 30 among children younger than 12 months, %CD4 < 25 among children aged 12–35 months, %CD4 < 20 among children aged 36–59 months; (3) patients had received ARV therapy for a minimum of 6 months prior to the study; (4) parents or guardians agreed to participate and provided written informed consent. Exclusion criteria were: (1) HIV-infected patients who failed therapy (stopped ARV therapy) or (2) patients who took any probiotic 2 weeks before or during the study period.

### Probiotic.

The microencapsulated *L. plantarum* IS-10506 (GenBank accession number DQ860148) powder was given 2.86 × 10^10^ cfu/day for 6 days. The probiotic powder was prepared under aseptic conditions, and in every stage of production, viability and contamination were assessed using plate counts in De Man, Rogosa and Sharpe (MRS) agar and Plate Count Agar (PCA), respectively.

### Blood lipopolysaccharide level.

The blood LPS level was measured pre- and post-treatment by ELI-SA using an Endotoxin Limulus Amoebocyte Lysate Assay (Thermo Scientific^™^ Pierce^™^ LAL Chromogenic Endotoxin Quantitation Kit, catalogue number 88282, Rockford, USA). For this procedure, the stock solution must be made using 1 mL of centrifuged blood serum. The blood LPS result was expressed in EU/mL. Finally, a conversion rate of 0.01 ng/mL of 0.1 EU/mL was used.

### Immunological analyses.

The absolute CD4+ T cell count, percent CD4, and absolute CD8+ T cell count were analysed by flow cytometry. Three millilitres of each blood samples with EDTA were analysed using flow cytometry (BD FACSCalibur, San Jose, USA) with BD Reagent (CD4 reagent catalogue No. 340383 and CD8 reagent catalogue No. 340344). Both the absolute count and percentage were obtained for the CD4+ and CD8+ T cell counts. The CD4+/CD8+ T cell ratio was obtained by calculation.

### Faecal secretory immunoglobulin A.

Stool samples were collected in a sterile stool container and immediately stored at −80°C. Faecal sIgA was determined using a solid phase direct ELISA sandwich method (Calbiotech, catalogue number SC221A, CA, USA). This method uses anti-human labelled horse-radish peroxidase (HRP) as the secondary antibody and tetramethylbenzidine (TMB) as the calorimetric substrate (both from Sigma-Aldrich, St. Louis, MO, USA). Results are reported in mg/gram stool.

### Statistical analysis.

Descriptive analysis was followed by independent t test and Mann-Whitney U test to compare pre-treatment and post-treatment groups and determine the differences between placebo and probiotic groups. Data were analysed using a 95% level of confidence (α = 0.05). Statistical analyses were done using SPSS 15.0 for windows (IBM, New York, USA).

## RESULTS

Twenty-five subjects met the inclusion and exclusion criteria and were randomly classified into the placebo group (n = 13) and probiotic group (n = 12). Four subjects, 2 subjects from the placebo group and 2 subjects from the probiotic group, discontinued participation owing to social reasons, such as distance of place of residence from Surabaya or inability to come for a required follow-up visit in 6 weeks. Therefore, a total of 21 subjects completed the study. Among these 21 subjects, 3 subjects were less than 5 years of age and 18 subjects were 5 years of age or older. Thus, based on WHO criteria, the percent CD4 criterion was applied to the 3 subjects aged < 5 years, while the absolute CD4+ T cell count criterion was applied to the 18 subjects aged ≥ 5 years.

There was no significant difference ( *p* > 0.05) in baseline characteristics, including the pre-treatment absolute CD4+ T cell counts, percent CD4, absolute CD8+ T cell counts, CD4+/CD8+ T cell ratio, and faecal sIgA level (Table 1); however, there was a significant difference ( *p* = 0.01) in the pre-treatment LPS level, which was higher in the probiotic group than in the placebo group. No serious side effects or other health issues, such as opportunistic infection, diarrhoea, or vomiting, were reported.

**Table 1.** Baseline characteristics

**Characteristic**	**Probiotic Group** **(n=10)**	**Placebo Group** **(n=11)**	***p***
**Age**			
< 5 years old	2	1	0.586^a^
≥ 5 years old	8	10	
**Sex**			
Male	4	7	0.395^a^
Female	6	4	
**Nutritional Status**			
Normal	9	11	0.476^a^
Poor	1	0	
**Place of Residence**			
Surabaya	6	6	1.000^a^
Outside Surabaya	4	5	
**Length of ARV therapy**			
≤ 1 year	0	0	N/A
> 1 year	9	11	
**LPS level pre-treatment, mean (SD) pg/mL**	130.00 (32.66)	95.45 (22.96)	0.011^*,c^
**CD4+ T cell absolute count pre-treatment, mean (SD) cells/mm^3^**	1023.50 (571.10)	995.50 (382.40)	0.903^c^
**%CD4+ T cell pre-treatment, mean (SD)**	34.06 (17.74)	23.97 (N/A)	0.723^c^
**CD8+ T cell absolute count pre-treatment, mean (SD) cells/mm^3^**	1594.70 (554.46)	1821.73 (601.45)	0.381^c^
**CD4+/CD8+ T cell ratio pre-treatment, mean (SD)**	0.77 (0.53)	0.61 (0.30)	0.622^b^
**Faecal sIgA pre-treatment, mean (SD) mg/g stool**	259.71 (28.91)	302.53 (52.21)	0.032^*,c^

a tested using Fisher's exact test

b tested using Mann-Whitney U test

c tested using independent t-test

* significant difference on (*p* < 0.05)

N/A: not analysed

**Fig. 1.**
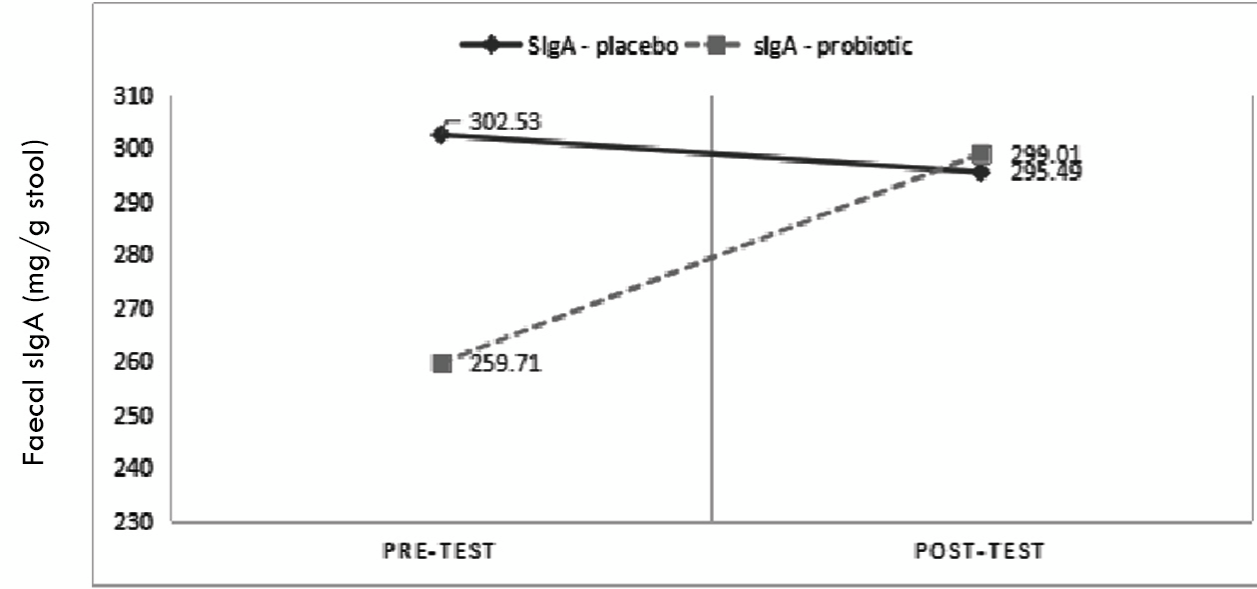
Comparison of faecal sIgA level (mg/g stool) between *L. plantarum* IS 10506-treated and placebo groups

**Fig. 2.**
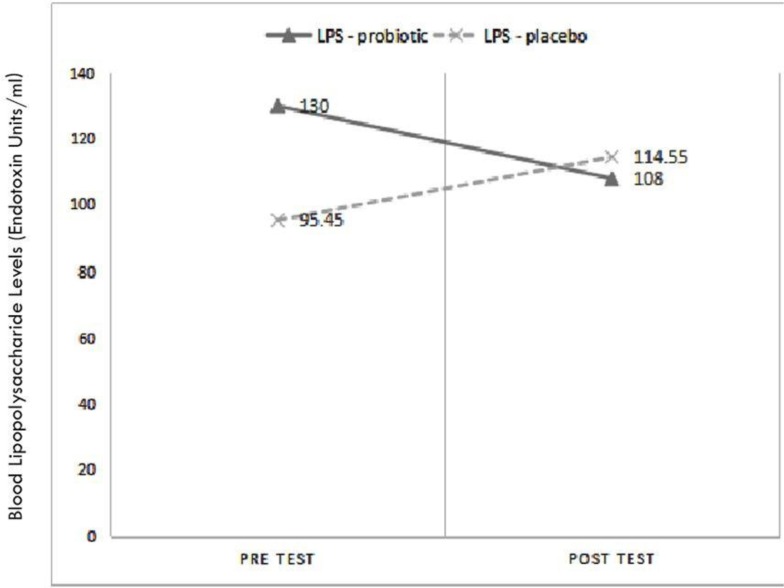
Comparison of blood LPS Level(EU/ml) between *L. plantarum* IS 10506-treated and placebo groups

We conducted statistical analysis based on the difference in the pre- and post-treatment LPS level (ΔLPS), absolute CD4+ T cell count (ΔCD4+), percent CD4 (Δ%CD4), absolute CD8+ T cell count (ΔCD8+), CD4+/CD8+ T cell ratio (ΔCD4+/CD8+), and faecal sIgA level (ΔsIgA). We found a significant reduction in the LPS level ( *p* = 0.011) in the probiotic group, whilst there was no significant difference in faecal sIgA, absolute CD4+ T cell count, percent CD4, absolute CD8+ T cell count, and CD4+/CD8+ T cell ratio between the probiotic and placebo group (Table 2). Interestingly, 6 weeks after intervention, the faecal sIgA level of probiotic group was significantly higher in the placebo group (Fig. 1). The post-treatment LPS level was lower than the pre-treatment faecal sIgA level in the probiotic group. However, there was a lower LPS level in the post treatment placebo group (Fig. 2).

**Table 2.** Pre- and post-treatment differences between the probiotic and placebo groups

	**Probiotic Group**		**Placebo Group**		***p***

	**n**	**Mean (SD)**	**n**	**Mean (SD)**	
ΔLPS level, pg/mL	10	−22.00 (24.86)	11	19.09 (23.00)	0.001^a,*^
ΔCD4+ T cell absolute count, cells/mm^3^	8	4.50 (178.99)	10	12.50 (344.88)	0.953^a^
Δ%CD4+ T cell	2	2.91 (5.18)	1	1.68 (N/A)	0.878^a^
ΔCD8+ T cell absolute count, cells/mm^3^	10	−108.20 (346.63)	11	−7.36 (519.30)	0.611^a^
ΔCD4+/CD8+ T cell ratio	10	0.0280 (0.1452)	11	0.0073 (0.0782)	0.684^a^
ΔFaecal sIgA, mg/g stool	10	39.30 (28.10)	11	−7.04 (57.96)	0.057^b^

a tested using independent t test

b tested using Mann-Whitney U test

* significant difference on (*p* < 0.05)

NA: not analysed

## DISCUSSION

The development of highly active antiretroviral therapy (HAART) has turned HIV infection into a chronic disease. Virological suppression and CD4+ T cell counts increment effect can be obtained for 12 months following therapy. One of the target organs of HIV infection is the gastrointestinal tract (14). In the immunopathogenesis of HIV, enteropathy often occurs in HIV-infected patients (15). Enteropathy can cause microbial translocation from the intestinal lumen to the systemic circulation (16). In normal conditions, microbes and their products in the gastrointestinal lumen will be phagocytosed in the lamina propria and mesenteric lymphatic circulation (6). However, in immunocompromised patients, this defence mechanism is dysfunctional.

Prior research has noted subtle decreases in IgA-producing plasma cells in the colon and duodenum of HIV-positive subjects relative to non-HIV subjects, which is thought to allow bacteria to translocate from the gut lumen into the systemic blood circulation (6). This translocation activates chronic systemic immune activation and persistent inflammation (17). In this study, we examined the blood LPS level to measure bacterial translocation (6), the faecal sIgA level to indicate the humoral mucosal immune response, and the absolute CD4+ T cell count, percent CD4, absolute CD8+ T cell count, and CD4+/CD8+ T cell ratio to measure systemic immune response. Our results showed that probiotics can decrease the blood LPS level in HIV-infected children undergoing ARV therapy, but they do not appear to influence the humoral mucosal immune response and systemic immune response.

Lipopolysaccharide is the main component of the Gram-negative bacteria cell wall and a potent inflammatory stimulant, which is present in measurable quantities in blood plasma. The lipopolysaccharide level can be observed as a consequence of gastrointestinal tract damage in patients with chronic infection, including those with HIV infection (4). Administration of ARV in patients with HIV infection has been known to suppress virus replication. However, evidence has shown that ARV alone does not help repair structural damage of the gastrointestinal tract, which is the main cause of bacterial translocation (14). In this study, supplementation with the probiotic *L. plantarum* IS-10506 led to a significantly lower blood LPS level in HIV-infected children who received ARV therapy.

Gori (2012) revealed that probiotic supplementation reduces microbial translocation. In this study FOS + *Lactobacillus rhamnosus* HN001 and *Bifidobacterium lactis* Bi-07 were administered to 20 adults for 16 weeks and showed decreased microbial translocation as measured by bacterial 16S rRNA, and decreased pathogenic bacterial count in HIV-infected adults who had not received ARV therapy (1). This study is similar to work by Hunt (2010) that used prebiotics as interventional treatment that led to a decrease in both LPS and the pathogenic bacterial count after 12 weeks of intervention in 20 adults who had not received ARV therapy (16). Furthermore, another study observing synbiotic administration for 4 weeks in 28 HIV-infected women who had received ARV therapy and a study which observed probiotic and prebiotic administration in SIV-infected Asian macaques given ARV therapy did not show a change in microbial translocation nor pathogenic bacterial count (8, 18).

Various studies have shown that probiotics play a role in reducing epithelial permeability and down-regulating systemic and mucosal inflammation (19). The epithelial brush border tight junction is damaged during infection. *L. plantarum* IS-10506 and IS-20506 helped repair intestinal epithelial brush border damage due to *E. coli* lipopolysaccharide. Probiotic administration has been shown to repair such damage, marked by a simultaneous increase in *Galectin-4, Myosin-1a, Occludin* and *ZO-1* through the ER1K/MAP (extracellular regulated kinase/mitogen activated protein) and JNK/MAP kinase (c-Jun NH2 terminal kinase/mitogen activated protein) pathways (12, 18, 20). Thus, the observed decrease in microbial translocation in our study could be caused by a direct repair mechanism affecting the mucosal barrier tight junction and a decrease in gastrointestinal epithelial permeability (20).

On the contrary, previous research suggests that intestinal barrier dysfunction may be related to the sIgA level (21). However, in our study, sIgA did not show a significant increase that correlated with the significant decrease in LPS level. Thus, we concluded that the probiotic reduces the LPS level through a pathway not involving sIgA.

Furthermore, this study also showed that the probiotic did not affect either the humoral mucosal immune response or systemic immune response in HIV infected patients. Hummelen (2010) studied the effect of probiotic supplementation containing *L. rhamnosus* GR-1 and *L. reuteri* RC-14 (2×10^9^ cfu) for 25 weeks on adult women with HIV infection. The probiotics did not affect immune system function (15). Experimental research on pigtail macaques given VSL#3 probiotic supplementation and ARV therapy showed that there was no difference in the CD4+ T cell content of blood, although there was an increase in the CD4+ T cell counts in the digestive system ( jejunum and colon) (8). Probiotic supplementation with ARV therapy was found to increase the CD4+ lymphocyte count significantly in the propria lamina cells in the small intestine of mice (8). Unfortunately, we did not measure the intestinal CD4 level.

There was no report of adverse effects or severe clinical changes during our study. Evidence showed that administration of prebiotics, probiotics, and synbiotics to immunocompromised patients due to HIV-infection did not produce significant adverse effects (20, 22). A previous study by Cunningham et al. showed that one-month administration of high dose *L. plantarum* 299v to HIV-infected children did not yield adverse effects (23). It was reported that prebiotics given to HIV-infected adults for 12 weeks cause only minor stomach discomfort and flatulence in a minority of subjects (1). A similar result was also reported in a study by Hernandez et al. using a synbiotic for 16 weeks in HIV-infected adult patients (20). The study showed no observable serious adverse effects, significant clinical changes, or occurrence of sepsis, bacteraemia, or an acute inflammatory response (20).

## CONCLUSION

From this study, we conclude that the probiotic *L. plantarum* IS-10506 can decrease the blood LPS level in HIV-infected children undergoing ARV therapy after 6 weeks of supplementation and has potential for use as a supplement for HIV-infected children who received ARV therapy. Further study is needed to specifically prove the role of probiotics in controlling microbial translocation, repairing the gastrointestinal mucosal barrier, and reducing gastrointestinal permeability, as well as to investigate the relationships among these factors.
